# The emerging health impact of voluntary medical male circumcision in Zimbabwe: An evaluation using three epidemiological models

**DOI:** 10.1371/journal.pone.0199453

**Published:** 2018-07-18

**Authors:** Jessica B. McGillen, John Stover, Daniel J. Klein, Sinokuthemba Xaba, Getrude Ncube, Mutsa Mhangara, Geraldine N. Chipendo, Isaac Taramusi, Leo Beacroft, Timothy B. Hallett, Patrick Odawo, Rumbidzai Manzou, Eline L. Korenromp

**Affiliations:** 1 Department of Infectious Disease Epidemiology, Imperial College London, London, United Kingdom; 2 Avenir Health, Glastonbury, Connecticut, United States of America; 3 Institute for Disease Modeling, Seattle, Washington, United States of America; 4 Ministry of Health and Child Welfare, Harare, Zimbabwe; 5 Zimbabwe National AIDS Council, Harare, Zimbabwe; 6 Independent consultant, Nairobi, Kenya; 7 Zimbabwe Country Office, Clinton Health Access Initiative, Harare, Zimbabwe; 8 Avenir Health, Geneva, Switzerland; NPMS-HHC CIC / LSH&TM, UNITED KINGDOM

## Abstract

**Background:**

Zimbabwe adopted voluntary medical male circumcision (VMMC) as a priority HIV prevention strategy in 2007 and began implementation in 2009. We evaluated the costs and impact of this VMMC program to date and in future.

**Methods:**

Three mathematical models describing Zimbabwe’s HIV epidemic and program evolution were calibrated to household survey data on prevalence and risk behaviors, with circumcision coverage calibrated to program-reported VMMCs. We compared trends in new infections and costs to a counterfactual without VMMC. Input assumptions were agreed in workshops with national stakeholders in 2015 and 2017.

**Results:**

The VMMC program averted 2,600–12,200 infections (among men and women combined) by the end of 2016. This impact will grow as circumcised men are protected lifelong, and onward dynamic transmission effects, which protect women via reduced incidence and prevalence in their male partners, increase over time. If other prevention interventions remain at 2016 coverages, the VMMCs already performed will avert 24,400–69,800 infections (2.3–5% of all new infections) through 2030. If coverage targets are achieved by 2021 and maintained, the program will avert 108,000–171,000 infections (10–13% of all new infections) by 2030, costing $2,100–3,250 per infection averted relative to no VMMC. Annual savings from averted treatment needs will outweigh VMMC maintenance costs once coverage targets are reached. If Zimbabwe also achieves ambitious UNAIDS targets for scaling up treatment and prevention efforts, VMMC will reduce the HIV incidence remaining at 2030 by one-third, critically contributing to the UNAIDS goal of 90% incidence reduction.

**Conclusions:**

VMMC can substantially impact Zimbabwe’s HIV epidemic in the coming years; this investment will save costs in the longer term.

## Introduction

Zimbabwe has mounted a strong response to its generalized HIV epidemic [1–3]. Average national HIV prevalence declined from 26.5% in 1997 to 14.3% in 2009 among adults ages 15–49 [4] in association with, and possibly accelerated by, reductions in risky sexual behavior [5, 6]. By 2016 the national HIV prevalence was 14% in adults (age 15–49) with 1.3 million people of all ages living with HIV and an estimated 60% of these virally suppressed on treatment, according to a national population-based HIV impact assessment (PHIA) [7].

Voluntary medical male circumcision (VMMC) has been established as an effective and efficient intervention against the spread of HIV; three randomized clinical trials in the early 2000s showed that it reduces the risk of female-to-male HIV transmission by 60% [8–10], and the protective effect is lifelong [11]. Recent meta-analyses have estimated this heterosexual risk reduction to be 70–72% [12, 13], with a reduction in male-to-male transmission risk of 20% [13]. Minimally invasive techniques are bringing costs down and expanding accessibility beyond clinics [14]. The US President's Emergency Plan for AIDS Relief (PEPFAR) began funding VMMC initiatives in sub-Saharan Africa in 2007. Subsequently, the World Health Organization (WHO) and Joint United Nations Program on HIV/AIDS (UNAIDS) set a target of reaching 80% VMMC coverage by 2015 among adult men (ages 15–49 years) in 14 priority countries with high HIV prevalence and historically low male circumcision (MC) rates in eastern and southern Africa [15], including Zimbabwe.

Zimbabwe adopted VMMC as a priority HIV prevention strategy in 2007 and began service delivery in 2009. Its initial plan was to reach 80% coverage among young men (ages 15–29) by 2017. Pilot studies and modeling of expected impact and cost [16–18] informed the strategy, design, and programmatic targets for VMMC scale-up between 2009 and 2015. Despite successes in service provision, uptake was slower than expected (with 845,000 VMMCs reported by 2016), possibly as a result of negative perceptions of circumcision in a traditionally non-circumcising country [19]. A bottleneck assessment in 2013 recommended initiatives such as focusing funds on community education and mobilizing women for demand creation [20]. A modeling study indicated that the program was reaching an efficient and impactful age bracket (13–29 year-olds) via school-based campaigns but boosting uptake among already sexually active young men would increase short-term impact and cost-effectiveness [21]. However, another study [22] cautioned that prioritizing older men (age 20 and over) might incur greater demand creation costs. 'Hotspots' of HIV risk identified in Zimbabwe informed the targeting of VMMC and other prevention efforts [23].

In 2016 the original targets were revised using a simple linear projection model, the Decision Makers' Program Planning Tool (DMPPT) [18]. The program’s ‘scale-up’ phase—the period until reaching the target coverage levels of 80% of 15–29 year old men and 30% of 10–14 year-olds—was extended to 2021, and corresponding numerical targets were developed for all districts. As the Zimbabwe VMMC program strives to reach these 2021 targets in a context of limited resources and competing intervention options, it is timely to evaluate its ongoing performance in terms of health and economic impact and cost-effectiveness. Here we present results from dynamic mathematical modeling of HIV transmission, epidemic spread, and interventions in Zimbabwe, to inform future decisions about the importance of the VMMC program for achieving epidemic control. This study was conducted jointly by three modeling groups, in consultation with national program planners and stakeholders, to produce robust consensus estimates of the historical and future impact of the Zimbabwe VMMC program. Our aim in pursuing a consensus was to confirm that the key findings were robust features of the epidemiological impact of VMMC, rather than artefacts arising from particular assumptions underpinning a single model.

## Methods

### Structure and calibration of the three mathematical models

Avenir Health applied the Spectrum Goals model [24], which has previously informed national strategic planning for HIV/AIDS in Zimbabwe [1] and other southern African countries (for example, [25]); Imperial College London (ICL) applied a risk-structured compartmental model of sexual HIV transmission among adults ages 15–49 [26]; and the Institute for Disease Modeling applied the EMOD-HIV individual-based model with explicit age structure (more details in Section A in S1 File).

All models independently projected the historical, ongoing, and future HIV epidemic in Zimbabwe: the Goals and ICL models for each of Zimbabwe’s ten provinces, and EMOD for the country as a whole but additionally fitting age-specific behavioral and epidemic outcomes (see Section B in S1 File).

All modeling groups agreed on key assumptions for HIV interventions, including that VMMC reduces the female-to-male transmission probability per sex act by 60%, as a conservative assumption [8–10, 12, 13]. This is the direct protective effect; additionally, as all three models are dynamic, the results include indirect effects on women via reduced incidence and prevalence in their male partners. We set the unit cost of VMMC at $109 per procedure, as a weighted average between surgical methods (forceps-guided or dorsal split) and the Prepex device [27]; between urban and rural locations; and between dedicated VMMC delivery points, integrated clinics, and outreach. The unit cost of ART was set at $251 per adult per year, including drugs, laboratory costs, and service delivery. This was based on a comprehensive estimate from a multiple-facility costing study in Zambia [28], as Zimbabwe does not currently have an official ART unit cost estimate, and we believe that the Zambian unit cost estimate should be a close proxy for Zimbabwe, with minor variations likely to be for inputs such as Human Resources.

### VMMC delivery and coverage, 2009–2016

The prevalence of traditional male circumcision in 2008 (just before the VMMC program) was determined from self-reported MC rates in Demographic and Health Surveys (DHS) from 2005/6 [29] and 2010/11 [30]. Some provinces had a lower self-reported MC rate in 2010 than in 2005, probably due to reporting, recall, or measurement errors. For our models, we set the 2008 MC rate to the lower of the two DHS estimates in each province (Table A in S2 File).

Numbers of VMMCs performed by age stratum were taken from client intake records at facilities for 2009–2014 and from the National District Health Information System [31] for 2015 and 2016 (Table B in S2 File). In the EMOD model, these VMMCs were distributed over time directly to male individuals of the appropriate age. The ICL model distributed VMMCs directly to the 15–49 age group, with boys in the 10–14 age group entering the model as newly circumcised with a delay of 5 years to simulate the onset of sexual maturity. For the Goals model, VMMC numbers were translated into proportions of adult men newly circumcised, by dividing the VMMCs by the corresponding male target population (by age stratum), adding that percentage coverage to the baseline (2008) MC coverage percentage, and subtracting MC coverage among men aging out of the sexually active group. This approach implicitly (and optimistically) assumes that there is no replacement of traditional MCs by VMMCs.

Although it may be beneficial to include HIV-positive males in VMMC programs [32], Zimbabwe’s present policy recommends circumcising HIV-uninfected men only. EMOD and ICL thus assumed that all VMMCs went to HIV-negative men, while the Goals model did not apportion VMMCs separately between HIV-positive and HIV-negative men.

### VMMC scale-up scenarios

Our counterfactual throughout is a 'no VMMC ever' scenario; that is, we assume that there was/is never a VMMC program in Zimbabwe, and MC coverage instead remains constant at the 2008 level in each province (Table 1).

**Table 1:** VMMC scale-up scenarios evaluated.

Scenario	Description
**No VMMC ever**	• Counterfactual scenario • Non-VMMC male circumcision coverage constant at 2008 level
**Program ends after 2016**	• Program VMMCs from 2009 through 2016 only • ART scaled up over 2017–2020 according to 2014–2016 trend and constant after 2020 • Prevention interventions at constant coverage and risk and protective behaviors constant from 2016 onward
**Program targets through 2021 met and maintained in a 'status quo' context**	• Program VMMCs from 2009 through 2016, program targets met by 2021, and maintenance of target coverage thereafter • ART scaled up over 2017–2020 according to 2014–2016 trend and constant after 2020 • Prevention interventions at constant coverage and risk and protective behaviors at constant rates from 2016 onward
**Program targets through 2021 met and maintained in a 'Fast Track' context**	• Program VMMCs from 2009 through 2016, program targets met by 2021, and maintenance of target coverage thereafter • ART and prevention interventions scaled up according to ambitious UNAIDS global Fast Track targets for 2021 and 2030 (Table A in S1 File)
**VMMC program ends after 2018/19**	• Program VMMCs from 2009 through 2016, program targets met up to 2018 (in 9 Global Fund-supported districts) or 2019 (in 36 PEPFAR-supported and 18 Bill & Melinda Gates Foundation-supported districts); no new VMMCs in any of Zimbabwe’s 63 districts after current (as of June 2017) funding commitments end • ART scaled up over 2017–2020 according to 2014–2016 trend and constant after 2020 • Prevention interventions at constant coverage and risk and protective behaviors at constant rates from 2016 onward

Our first scale-up scenario considers the program up to 2016 only; that is, the models capture the VMMCs performed over 2009–2016 (described above), but assume that no further VMMCs would be performed after 2016.

Our second scale-up scenario supposes that the VMMC program targets—scale-up to 80% coverage among 15–29 year old men and 30% among 10–14 year-olds—are achieved by 2021 and maintained thereafter. We evaluate this scenario in two possible settings, which capture developments with other HIV interventions in Zimbabwe over the same time period. In a 'status quo' setting, the VMMC program could be accompanied by continuations of recent trends in antiretroviral therapy (ART) scale-up, with constant coverage of prevention interventions and constant rates of risk and (non-VMMC) protective behaviors. For the Goals model, this translates to extrapolating the trend in the proportion of people on ART from 2014–2016 [33] through 2020; the ICL model similarly extrapolates from historical numbers of adults on ART over 2004–2013 [34]; and EMOD mechanistically simulates the care continuum, in which increasing ART coverage stems from HIV testing and linkage into care. Alternatively, ambitious scale-up programs for both ART and prevention interventions could be enacted to reach UNAIDS Fast Track targets for 2030 [35], with the VMMC program playing a role as one of many initiatives in that setting (Section C in S1 File).

For an additional "pessimistic" model scenario, we assume that the funders of Zimbabwe’s existing program—PEPFAR, the Global Fund to fight AIDS, Tuberculosis and Malaria, and the Bill & Melinda Gates Foundation—discontinue district-level programs when their current commitments end in 2018/2019.

In the Goals model, ART reduces the HIV transmission probability per sex act by 80% in the 'status quo' scenarios (except for Bulawayo and Harare, for which the reduction is 75% according to best model fits), increasing to 90% in the Fast Track scenario in all provinces. In the ICL model, ART reduces the per-partnership transmission probability by 85% in all scenarios. EMOD simulates viral suppression from ART and assumes suppressed individuals are non-infectious.

### Evaluation design

Results for each model and scenario were evaluated and compared for: new HIV infections, numbers of VMMC conducted, cost of VMMC and cost of ART, and the resulting number of VMMCs or cost per infection averted, relative to the counterfactual scenario without the VMMC program. We did this in turn for multiple timeframes, looking either historically (over 2009–2016) or into the future (projecting forward from 2017 through 2030). In the Results section, the presented ranges reflect differences in parameters and structure between the three models (Section D in S1 File).

## Results

### VMMC scale-up

**Fig 1:**
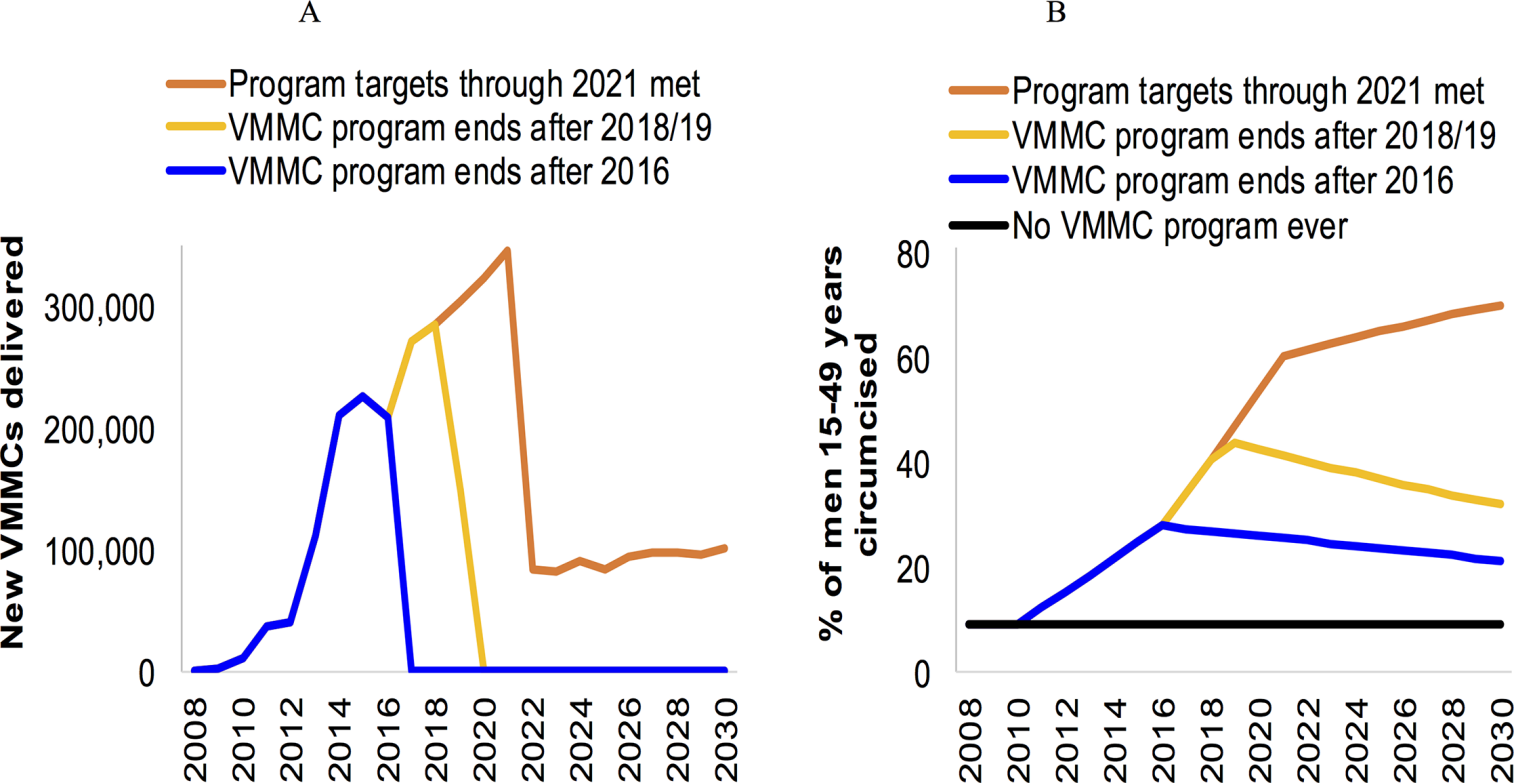
Modeled circumcisions. **(A) Number of new VMMCs occurring each year and (B) the resulting percentage of men ages 15–49 who are circumcised, by scenario.** The 2009–2016 new VMMCs are from program data; the 2008–2009 circumcision coverage was the modelers’ estimate based on the 2005 and 2010 DHS. The projected results (VMMC numbers and circumcision coverage over 2010–2030) shown here are from the Goals model; the ICL and EMOD models projected similar numbers (see Table 2) and coverages (not shown).

Each model depicts the historic and targeted VMMC scale-up patterns slightly differently, due to differences in model structure, parameters and demographic inputs. However, the patterns of VMMCs achieved in the past and targeted for the future (Fig 1A; details in S3, S4 and S5 Files) and resulting MC coverage over time (Fig 1B, e.g. 28% in 2016) are broadly in agreement with the program data and targets [27].

If the program targets for 2017–2021 are achieved, the projected annual VMMCs drop sharply from 2022, once the program's 'scale up' phase has been completed and the coverage targets are reached. During the subsequent 'maintenance' phase, new VMMCs are needed only among the small number of boys who age into the sexually active cohort each year. The resulting MC coverage (comprising these few new VMMCs and the time-constant coverage of traditional circumcision at 9.2% among men ages 15–49) continues to increase over 2022–2030.

In our scenarios in which no future VMMCs are performed (i.e. the program ended in 2016) or the program is halted in 2018/19 as current funder commitments expire, coverage in the 15–49 year old cohort falls gradually back to the baseline level of 9.2% as uncircumcised 14-year-old boys age into the cohort and circumcised 49-year-old men age out.

### Impact of the VMMC program, 2009–2016

Across the three models, the VMMC program has averted an estimated 2,600–12,200 HIV infections over 2009–2016. This has required 69–335 circumcisions or a cost of $7,600-$36,500 to avert one new infection (Table 2).

**Table 2:** Projected impact, costs and savings from the VMMC program in a 'status quo' background context, relative to the counterfactual scenario of no VMMC program ever.

Scenario	Outcome	Goals model^a^	ICL model^b^	EMOD model^a^^,^^c^
Results over 2009–2016	Number of VMMCs performed	845,500	845,428	891,500
	Number of HIV infections averted	12,200 (2%)	7,200 (1.6%)	2,600 (0.5%)
	Number of VMMCs per infection averted	69	115	335
	Cost per infection averted	$7,600	$12,600	$36,500
Results 2009–2030; scenario Program ends after 2016	Number of VMMCs performed	845,500	845,428	891,500
	Number of HIV infections averted	69,800 (5%)	24,400 (2.3%)	52,250 (4%)
	Number of VMMCs per infection averted	12	34	17
	Cost per infection averted	$1,320	$3,700	$1,860
Results 2009–2030; scenario Program ends after 2018/19	Number of VMMCs performed	1,777,000	1,515,900	1,262,000
	Number of HIV infections averted	126,000 (10%)	37,500 (3.5%)	71,000 (6%)
	Number of VMMCs per infection averted	14	40	18
	Cost per infection averted	$1,500	$4,400	$1,900
Results 2009–2030; scenario Program targets through 2021 met and maintained	Number of VMMCs performed	3,257,000	3,158,000	3,210,000
	Number of HIV infections averted	171,000 (13%)	128,000 (12%)	108,000 (10%)
	Number of VMMCs per infection averted	19	25	30
	Cost per infection averted	$2,100	$2,700	$3,250
	Savings in ART costs, 2017–2030	$198 million	$55 million	$158 million

The percentages in parentheses represent the proportion of new HIV infections averted relative to the number of new HIV infections in the counterfactual scenario.

^a^ Health and cost-effectiveness outcomes are for all ages

^b^ Health and cost-effectiveness outcomes are for age 15–49 years only

^c^ Over this short time period, EMOD outcomes are influenced by noise from stochastic variation.

### Long-term impact of VMMC in a 'status quo' context

**Fig 2:**
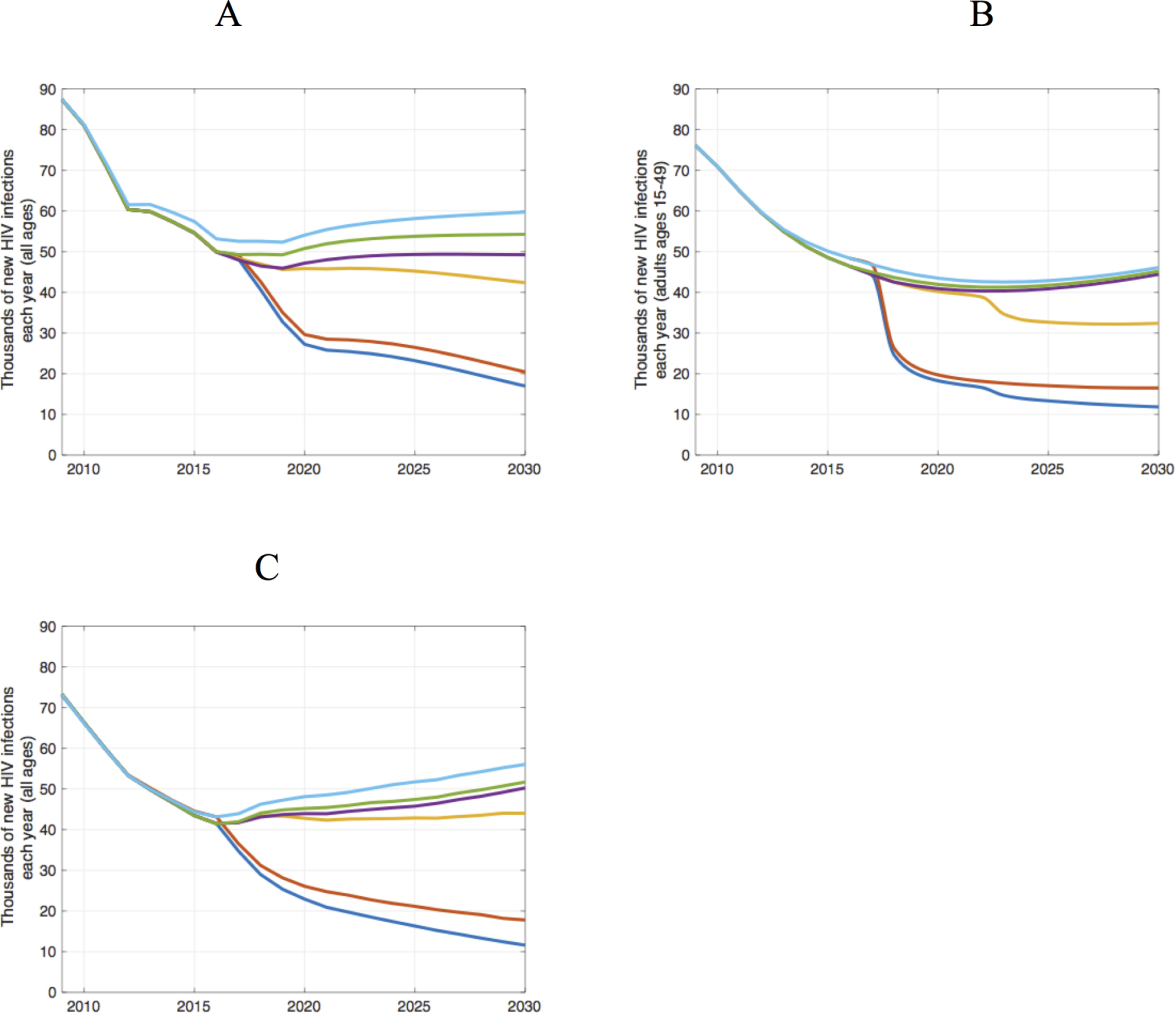
**Number of new HIV infections each year over 2009–2030 by scenario, produced by the (A) Goals, (B) ICL, and (C) EMOD models.** The scenarios shown are: the counterfactual of no VMMC ever (turquoise), the VMMC program ends after 2016 (green), the VMMC program ends after 2018/19 (purple), the program targets through 2021 are met and maintained in a 'status quo' context (yellow), Fast Track targets are achieved without VMMC (red), and the VMMC program targets are achieved in a Fast Track context (blue). Infections are among all ages in the Goals and EMOD models (A and C, respectively) and among ages 15–49 in the ICL model (B).

Against a 'status quo' backdrop of continued ART scale-up, we evaluated the impact of VMMC over the longer timeframe of 2009–2030 (Table 2 and Fig 2). The VMMCs provided up to 2016 will avert 24,400–69,800 new infections (2.3–5% of all new infections) over 2009–2030, even if no further VMMCs are performed. Infections averted after 2016 result primarily from young men circumcised between 2009 and 2016, who age into their life stage of highest HIV incidence in next years.

Achieving the VMMC targets by 2021, with maintenance thereafter, will avert 108,000–171,000 infections (10–13% of all new infections) over 2009–2030. VMMC is an efficient intervention, requiring only 19–30 circumcisions per infection averted. Sustaining the accelerated VMMC program will require continued funding and support. Indeed, the US President's Emergency Plan for AIDS Relief (PEPFAR), one of the main funders, plans to continue supporting VMMC programs in 14 countries and aims to achieve saturation by 2020 in priority districts [36]. However, recent changes in the political climate make it worth considering what might happen if funders withdraw their support. We estimate that if funders discontinue support for the VMMC program when their current commitments end in 2018/19, then 37,500–126,000 infections will be averted over 2009–2030, requiring 14–40 circumcisions per infection averted.

**Fig 3:**
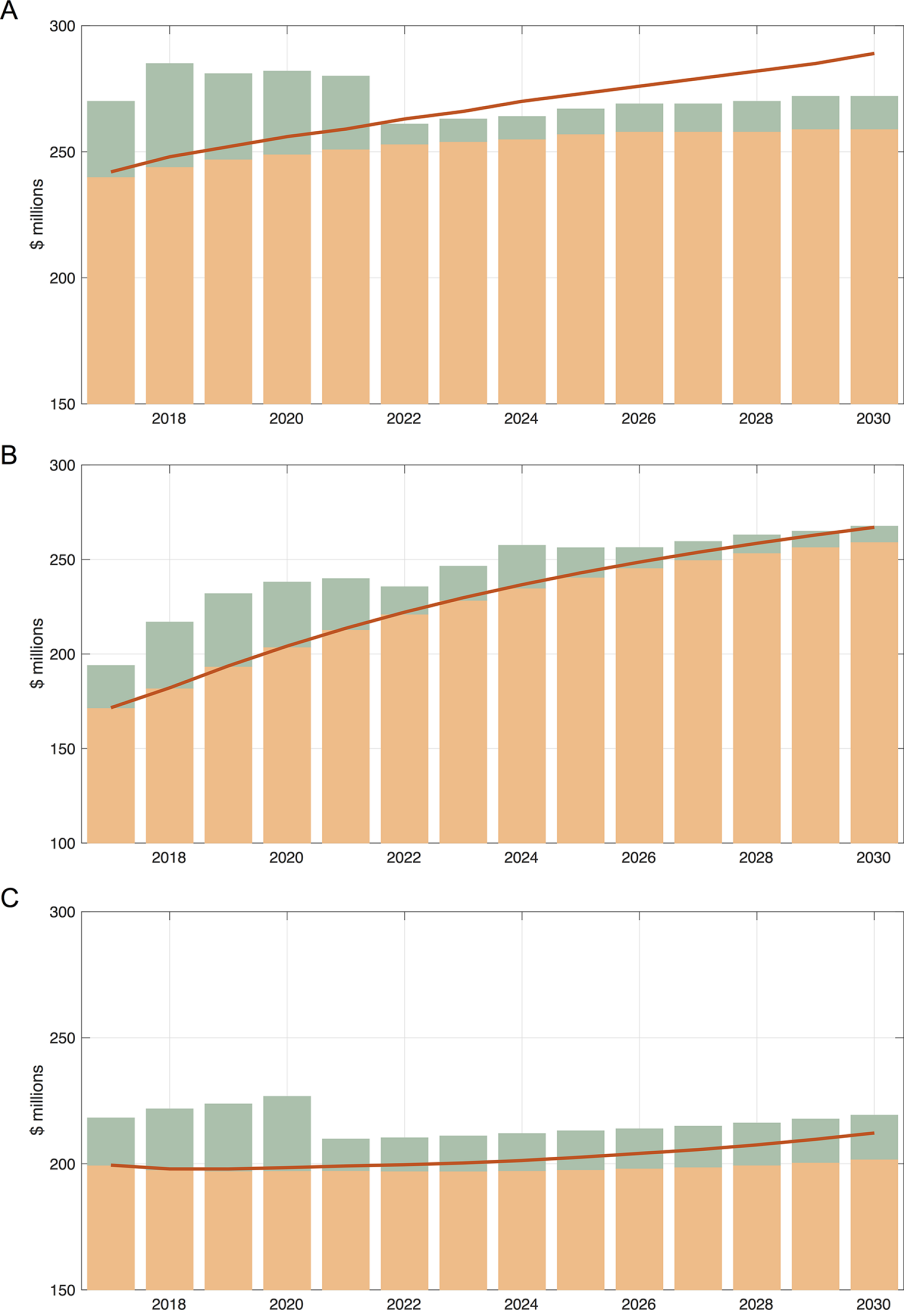
**Costs of VMMC and ART in the scenario in which the program targets for 2021 are achieved in a 'status quo' setting (without Fast Track), compared to our counterfactual scenario of no VMMCs ever, from (A) the Goals model, (B) the ICL model, and (C) the EMOD model.** Shown are the cost of VMMC if the program targets are met and maintained (green bars), the cost of ART if the program targets are met and maintained (orange bars), and the cost of ART in the counterfactual scenario with no VMMC ever (red curves). As in previous tables and Figs, Goals (A) and EMOD (C) consider all age groups and ICL (B) only ages 15–49.

While additional funds will be required to meet the VMMC target by 2021, after the target is reached, fewer annual resources will be required to maintain VMMC coverage. Over 2009–2030, the cost to avert one HIV infection with VMMC will be only $2,100–3,250. The models agree that once the target VMMC coverage levels have been reached, the net annual cost of maintaining treatment provision and the VMMC program together will be lower than what it would cost to maintain treatment in the absence of the VMMC program, because the aversion of new infections by VMMC will reduce the long-term treatment need. The 'break-even point' occurs in 2022 in the Goals projections, 2030 in the ICL projections, and 2035 in the EMOD projections (Fig 3). The reduction in net costs may reach $55–198 million over 2017–2030 (Table 2).

### Long-term impact of VMMC in the context of Fast Track

In the Fast Track scenario with accelerated scale-up of ART and additional prevention interventions alongside VMMC (Section C in S1 File), HIV incidence will be reduced to low levels, so that there are fewer remaining infections avertable by VMMC, and therefore the number of VMMCs needed to avert one infection and the cost to avert one infection are both higher (Table 3). Nevertheless, the marginal impact of VMMC is still substantial: new infections at 2030 under Fast Track would be up to 20% higher if VMMC were dropped from Fast Track and if MC coverage stayed at the 2016 level throughout 2030 (Fig 2). For example, according to the Goals model, we would see 19,600 infections in 2030 in a Fast Track setting without VMMC, compared to 16,100 infections in a Fast Track setting with VMMC.

**Table 3:** Projected impact of the VMMC program over 2016–2030, with VMMC scale-up embedded within broader scale-up of HIV prevention and treatment, according to the global Fast Track targets, evaluated relative to the counterfactual scenario in which the Fast Track targets for other interventions are met without VMMC.

Outcome	Goals^a^	ICL^b^	EMOD^a^
Number of infections averted	39,800	52,000	64,000
Number of VMMCs per infection averted	82	60	51
Cost per infection averted	$9,000	$6,560	$5,500

^a^ Outcomes are for all age groups

^b^ Outcomes are for ages 15–49 years only.

### Sensitivity analyses

We carried out a series of sensitivity analyses (Table 4). Thus far, we did not discount future outcomes; as a first sensitivity analysis, we applied a discounting of 3% per year to both future impacts and costs, back to 2015 as the reference year. Over 2009–2030, we found that discounting would increase the cost of VMMC per infection averted from $2,100–3,250 to $2,400–3,700, as the health impact lags behind the cost.

**Table 4:** Sensitivity analyses. Impact and cost-effectiveness of Zimbabwe’s VMMC program under alternative assumptions for key parameters whose values are uncertain.

Scenario	Infections averted			Cost^a^ per infection averted		
	Goals^b^	ICL^c^	EMOD^b^	Goals^b^	ICL^c^	EMOD^b^
**Default / best estimate**^d^	**171,000**	**128,000**	**108,000**	**$2,100**	**$2,700**	**$3,250**
HIV infections averted and costs discounted at 3% per year from 2015	131,000	96,000	81,000	$2,400	$3,200	$3,700
VMMC coverage at 2016 according to the 2015 DHS (14%) rather than the program estimate (28%)^e^	156,000	120,000	104,000	$2,200	$2,100	$3,300
Add VMMC protective efficacy reducing M-to-F transmission by 46%	287,000	Not evaluated	Not evaluated	$1,200	Not evaluated	Not evaluated
EMOD with VMMC targeted across the male population aged 15–49 years	NA / as default		101,000	NA / as default		$3,600

The results shown are for the scenario in which the program targets for 2021 are achieved in a 'status quo' context, with impact evaluated over 2009–2030.

^a^ Cost is direct VMMC program cost, not considering savings from ART averted

^b^ Outcomes are for all ages

^c^ Outcomes are for ages 15–49 only

^d^ Default assumptions: No discounting, 2016 VMMC coverage at 28% per program service delivery statistics, VMMC protective efficacy on female-to-male transmission 60% per sex act, no direct effect of VMMC on male-to-female transmission

^e^ See additional detailed results for other scale-up scenarios and time horizons in Section E in S1 File.

We also ran the models using an alternative estimate of 'baseline' (2016) MC coverage taken from the 2015 DHS and 2016 PHIA surveys rather than program data (see Section E in S1 File for a full discussion). With lower, survey-based MC coverage at 2016 (14% rather than 28%), impact is shifted to later years, and so the cost per infection averted is higher, especially for the shortest evaluation horizon up to 2016. However, for the scenario in which the 2021 program targets are achieved in a 'status quo' setting, the projected impact and cost-effectiveness over 2009–2030 are only 4–22% lower than the results under the default assumptions. This is because fewer VMMCs over 2009–2016 are required to achieve the survey-reported 14% national coverage by 2016, but these must then be compensated by more VMMCs projected over 2017–2021 to reach the program targets (Table 4). Additional results are provided in Section E in S1 File.

While the VMMC trials that underpinned the global recommendations for VMMC as a key HIV prevention strategy had evaluated protective effects on men only, later evaluations have demonstrated additional direct protective benefits for female partners of circumcised men. A modeling study of Zimbabwe and Kenya estimated that VMMC reduces the rate of male-to-female HIV transmission by 46% from two years after the procedure, in addition to the 60% reduction in the female-to-male rate [17]. When we add a 46% protection against male-to-female transmission, the impact in terms of infections averted is 68% higher, and cost-effectiveness about 50% better, than in the default results (Table 4).

Thus far we have assumed a constant ART unit cost of $251 per person per year, but in reality the cost may decline over time in response to antiretroviral price drops and improvements in the efficiency of service delivery. This has been predicted and targeted in the strategies of major global donors. If, for example, we allow a linear decline from $251 in 2017 to $200 by 2026, then the savings from averted ART costs due to VMMC over 2017–2030 are $47–178 million, rather than the default $55–198 million.

Finally, EMOD is the only age-structured model used here, and possible bias may arise from the lack of age structure in the Goals and ICL models. To evaluate this bias, the EMOD model was run without age targeting. Specifically, the annual coverage of VMMC in the population of 15–49 men resulting from age-targeted VMMC was recorded from the status quo program scenario. Then, the model was configured to reproduce this coverage level, but in the general 15–49 population by distributing VMMC to HIV-negative individuals in this group independently of their age. The results show a small increase (17.5%) in the number of infections averted and slightly improved cost per infection averted over 2009–2016, but a small reduction (7.5%) in infections averted and cost-effectiveness over 2009–2030 (Table 4). These effects reflect that short-term impacts depend on VMMC coverage in men in their 20s and 30s, whereas Zimbabwe’s VMMC program is reaching mostly adolescent boys, which is the strategy to achieve highest long-term impact. This EMOD variant thus suggests that Goals and ICL, by ignoring age structure, may have slightly over-estimated impact and cost-effectiveness in the short-run (2009–2016), but slightly under-estimated these in the longer-term.

## Discussion

Projections by our three independent models show that the Zimbabwe VMMC program will substantially impact the country’s HIV epidemic in the coming years. Cost per infection averted is low compared to most other HIV prevention interventions, as well as in comparison to key global non-HIV public health/infectious disease interventions (for example, [37], Table 2 in [38], and [39]). In addition, investing in VMMC will save costs in the longer term by reducing treatment need. VMMC is critical for achieving the Fast Track target of a 90% reduction in HIV incidence by 2030. In a wider context of concerns about plateauing global resources for HIV/AIDS and mounting long-term costs of ART programs, these are important findings to underpin continued high-priority investment in this uniquely cost-effective prevention intervention in Zimbabwe. Other countries with similar conditions—a generalized, high-prevalence HIV epidemic and low coverage of traditional circumcision—could benefit from prioritized investment in VMMC as well. It should be noted that our models projected clear benefits and returns on investment from VMMC even while also assuming a high effectiveness of ART, which itself reduces HIV infectivity and transmission.

The ranges for impact produced by the three models are fairly wide, reflecting real uncertainty in VMMC impact and its determinants, particularly the current level of HIV incidence. For example, differences between the two models without age structure (Goals and ICL) stem from the ICL model considering only ages 15–49 (versus all ages) and depicting a lower proportion of total incidence occurring among men than women, though both models are consistent with historical data. Additional uncertainty comes from the age distribution of incidence relative to the age distribution of new VMMCs. The age-structured EMOD model may, in this respect, have provided the best impact estimate, although data limitations make it difficult to fully validate modeled age structure in sexual networks. Nevertheless, our results are in line with earlier evaluations of VMMC cost-effectiveness in other sub-Saharan African settings; for example, VMMC was estimated to cost between $131 and $3,160 per infection averted across three regions in Mozambique [25], and $4,400 per infection averted as a median across 14 priority countries in eastern and southern Africa [40]. Cost per infection averted was somewhat lower in South Africa ($181–551) [41], likely because HIV incidence is very high there. The consistency across (past and current) modeling results provides credibility, especially given the distinct structures of our three models (Section D in S1 File).

Compared to projections used to inform Zimbabwe’s first VMMC program plan (ASCOP) in 2014 [27], the current models estimated fewer infections averted, a higher cost per infection averted, and a later time at which the savings from averted ART will begin to compensate for VMMC investments. One reason for this is that the program reported fewer VMMCs performed in 2015 and 2016 than the targets set out in the ASCOP, and the 80% coverage target was postponed from 2017 to 2021. Furthermore, HIV incidence as of 2016 is lower than what was projected in 2014, possibly reflecting an emerging impact from ART scale-up.

Our current analysis—and past cost-effectiveness analyses [22, 25, 40, 41]—have mostly focused on the direct benefits of VMMC for protecting HIV-negative men against acquisition from HIV-positive female partners, amplified by indirect effects on women through the reduced HIV prevalence among their male partners. However, the impact and cost-effectiveness become even more favorable if additional benefits are considered, such as direct protection of HIV-negative women against HIV acquisition from circumcised HIV-positive male partners (see our sensitivity analysis), the concurrent protection afforded by VMMC against sexually transmitted bacterial infections, and a side benefit of pre-VMMC HIV testing as a way to bring HIV-positive men into care.

### Limitations

Preliminary outputs from the 2016 PHIA [7] estimated HIV incidence among adults (ages 15–49) at 0.48 (0.29–0.66) per 100 person-years in 2016. Incidence levels generated by the three models for 2016 are 0.58/100 in Goals, 0.81/100 in the ICL model, and 0.65/100 in EMOD. While the Goals and EMOD models fall within the PHIA’s 95% confidence interval, the ICL model assumed a slightly higher baseline level of preventable infections. A modes-of-transmission data synthesis [4] put HIV incidence at 0.85 (0.56–1.17) per 100 person-years in 2009; estimates for 2009 from Goals, ICL, and EMOD were 1.37/100, 1.23/100, and 1.38/100, respectively. However, measuring HIV incidence directly with recent-infection assays, as in the PHIA, introduces substantial uncertainties related to accuracy, statistical power, and appropriate thresholds for defining "recent infections" (which vary between locations and over time). All three models were well-fitted to key data from both household surveys and routine sentinel clinic-based surveillance, with particular weight given to HIV prevalence and numbers of people on ART. Thus, while the relatively high modeled baseline incidence rates may have slightly inflated our estimates of absolute impact and cost-effectiveness, our results hold in terms of returns on VMMC investments in the longer term (through savings on ART)—and the critical importance of VMMC in a Fast Track context. Nevertheless, to refine models and VMMC impact projections in future, we recommend consideration of final PHIA incidence estimates and any other forthcoming independent incidence estimates.

Our ingredients-based unit cost for VMMC included national-level costs for demand creation but excluded the costs of monitoring and evaluation, provincial and district management and coordination, resource mobilization, and partner-level overheads [27]. This may mean that we have overestimated the cost-effectiveness of VMMC. Other uncertainties may arise from selective uptake; that is, we may have overestimated impact if VMMC clients are lower risk than average, or underestimated it if they are of higher risk. Market research in Zimbabwe in 2013 found that men taking up VMMC generally had attitudes suggesting low risk, but their actual behaviors were not measured [42]. Additionally, men might adopt riskier behaviors after circumcision (behavioral risk compensation), which would lead to overestimation of VMMC impact. However, a study of Bulawayo province in Zimbabwe over 2012–2015 found no indication of risk compensation [43], similarly to Kenya [44] and Uganda [45]. A further source of impact overestimation could be elevated risk during wound healing, but there is currently no evidence for or against this in Zimbabwe. Lastly, our modeling study did not aim to evaluate the possible impact of early infant male circumcision—which could be a useful and cost-effective strategy in the longer term, despite requiring a different implementation infrastructure—to supplement current adolescents- and adult-targeted strategies that are essential for short-term impact [46].

### Programmatic implications

Our consensus modeling results, together with identified uncertainties and data limitations, suggest actions that may help Zimbabwe's VMMC program to maximize its impact and cost-effectiveness, and strengthen future evaluations. In particular, uncertainty in the coverage of male circumcision achieved by 2016 (28% per program data versus 14% per 2015–16 national survey data; see Section E in S1 File) highlights the importance of strengthening monitoring of circumcision coverage coming from both VMMCs via programmatic service delivery statistics and traditional methods via population-based surveys.

### Concluding remarks

This consensus analysis shows the VMMC program in Zimbabwe has already had impact, and its health and economic benefits will grow significantly in the future. By averting infections, VMMC averts future costs of ART, such that prioritizing and investing in VMMC now will pay off in the longer term. VMMC is a key component of the UNAIDS Fast Track strategy, and will be critical for achieving the Fast Track targets for HIV incidence reduction and ending AIDS as a public health threat in Zimbabwe.

## Supporting information

S1 File **A Structures of the three mathematical models**.
**B Model calibrations to historical epidemics in Zimbabwe's 10 provinces**
Figure A: Goals model calibrations
Figure B: ICL model calibrations
Figure C: EMOD model calibrations
**C Assumptions for interventions in a 'Fast Track' modeling context**
Table A: Prevention and ART coverage target
**D Key model differences**
Table B: Comparison of model structures
**E Alternative estimate for baseline circumcision coverage in 2016**
Figure D: Alternative circumcision coverages among men ages 15–49
Table C: Impact over 2009–2016
Table D: Impact over 2009–2030
**References (continued from main text)**.
(DOCX)

S2 File Table A. Self-reported Male Circumcision status, in national household surveys.
Table B. Program-reported numbers of Voluntary Medical Male Circumcisions in Zimbabwe, by age group, province and year, 2009–2016; and resulting estimated proportion of men circumcised at 2016.
(XLSX)

S3 File Detailed annual results of the Goals model. (XLSX)

S4 File Detailed annual results of the ICL model. (XLSX)

S5 File Detailed annual results of the EMOD model. (XLSX)

## Abbreviations

ART: antiretroviral therapy
DHS: Demographic and Health Survey
PHIA: population-based HIV impact assessment (a national household survey conducted in 2016 in Zimbabwe)
VMMC: voluntary medical male circumcision
MC: male circumcision
