# Supplementary material for: The emerging health impact of voluntary medical male circumcision in Zimbabwe: An evaluation using three epidemiological models
Source: PLoS One. 2018 Jul 18;13(7):e0199453. doi: 10.1371/journal.pone.0199453 (PMC6051576; doi:10.1371/journal.pone.0199453)
Supplement: S1 File — A Structures of the three mathematical models. B Model calibrations to historical epidemics in Zimbabwe's 10 provinces Figure A: Goals model calibrations Figure B: ICL model calibrations Figure C: EMOD model calibrations C Assumptions for interventions in a 'Fast Track' modeling context Table A: Prevention and ART coverage target D Key model differences Table B: Comparison of model structures E Alternative estimate for baseline circumcision coverage in 2016 Figure D: Alternative circumcision coverages among men ages 15–49 Table C: Impact over 2009–2016 Table D: Impact over 2009–2030 References (continued from main text). (DOCX) [file pone.0199453.s001.docx]

The emerging health impact of voluntary medical male circumcision in Zimbabwe: an evaluation using three epidemiological models

*McGillen JB, Stover J, Klein DJ, Sinokuthemba X, Ncube G, Mhangara M, Chipendo GN, Taramusi I, Beacroft L, Hallett TB, Odawo P, Manzou R, Korenromp EL*

**Supporting Information file**

19 June 2018

# A Structures of the three mathematical models

The compartmental, risk-structured Goals model sits in the Spectrum platform, building on a demographic module that projects populations over time and models HIV epidemic spread between compartments of adults 15-49 years: low-risk adults who have one heterosexual partner; medium-risk with two or more partners in a year, high-risk adults who are female sex workers (FSW) and their clients, and men who have sex with men (MSM). Each group is characterized by numbers of partners, acts per partner per year, condom usage rate, and age at first sexual relationship, which can all be set to change over time, either as spontaneous social trends or in response to behavioral interventions; as well as proportion married (time-constant). The probability of HIV transmission is determined by type of contact, disease stage, MC status of male (uninfected) partners, condom usage, and ART status of the infected partner. Parameters determining the probability of HIV transmission are sampled from plausible ranges (established from literature) to optimally fit the historical epidemic. Adult HIV incidence and prevalence from Goals is fed into the linked Spectrum module AIM, which translates these into outputs such as numbers of people living with HIV (including pediatric infections), new infections, AIDS deaths, the need for ART, and prevention of mother-to-child transmission, by age group including those below 15 and above 49 years.

In the compartmental, risk-structured Imperial College London (ICL) model, risk groups among the age 15-49 population are female sex workers and their male clients (both very high risk), men who have sex with men (moderately high risk), men and women who form casual heterosexual partnerships (moderate risk), and men and women who tend to form stable long-term heterosexual partnerships (low risk). Older adults (ages 50+ years) are also in the model, but are assumed to no longer be at risk of HIV acquisition. The rate of HIV acquisition is driven by the force of infection, which is dependent on compartmental average HIV prevalence, disease stage, ART coverage, and sexual behaviors, including patterns of preferential sexual mixing within and between risk groups. Once infected, the model describes disease progression through a series of CD4 cell count stages [47], movement onto treatment, and prevention interventions which reduce the force of infection. Parameters governing sexual behaviors and demographics are calibrated within plausible ranges using a simplex search algorithm to maximize a standard likelihood expression.

The individual-based EMOD-HIV model describes HIV as transmitted primarily through age-structured heterosexual coital acts between individuals in lasting relationships of informal, marital, and commercial types. Each individual has a date of birth, from which age is computed, and is subject to detailed vital dynamics. Infectivity of HIV+ individuals depends on infection stage (acute, latent, AIDS), log-normally-distributed individual-level heterogeneity [48], viral suppression due to ART, STI co-infection, condom usage, and more. Male susceptibility is reduced by VMMC on a per-coital-act basis. While most of the population is serially monogamous, medium-risk individuals can have concurrent relationships and high-risk female sex workers are at high risk of participating in HIV transmission with their clients. HIV testing occurs voluntarily, at antenatal visits, or once symptomatic. Individuals flow through a realistic representation of the ART continuum to access treatment, and may discontinue and later resume care. Of the three models in this study, EMOD is the only model to explicitly capture age structure. Bayesian history matching [49] was used to identify 100 unique parameter combinations that are not inconsistent with historical data. Each parameter combination was replicated 10 times for each modeling scenario to reduce stochastic noise.

# B Model calibrations to historical epidemics in Zimbabwe's 10 provinces

Each of the three models was independently calibrated to historical data on Zimbabwe's HIV epidemics. The Goals and ICL models were calibrated to data on each province, while the EMOD model was calibrated to national-level data but additionally accounting for age-specific outcomes. For all models, fertility and mortality were set according to census data and estimates from the 2015 UN World Population Prospect (WPP) [50]. Key epidemic data for model calibration were sero-prevalence estimates in adult men and women from demographic and health surveys in 2005/6 [25], 2010/11 [26], 2015 [51], and 2016 [7], as well as time trends in prevalence from sentinel surveys of antenatal clinics. Historical patterns in risk behaviors and condom usage, which determine historical HIV transmission in the models, were informed by data from the same surveys and epidemiological, behavioral, and intervention studies in Zimbabwe (e.g. [52, 53])—notably considering the importance of structural and socio-demographic factors, including sexual networks with concurrent partnerships such as 'small houses' (men having stable partnerships alongside marriage) [54]. HIV transmission probabilities and disease progression rates were fitted, within plausible ranges established by longitudinal studies in the South and East African region, to produce the best fit in terms of HIV prevalence by province (Goals and ICL), by age (EMOD), and by gender (all models). Numbers of adult men, adult women, and (in Goals) children under 15 years living with HIV who were on ART over 2000-2016 were taken from national HIV program statistics. The resulting calibrated models are shown in Figure A (Goals), Figure B (ICL), and Figure C (EMOD).

**Figure A. Goals model calibrations** to the median (solid curve), maximum and minimum (dashed curves) model outputs for percent HIV prevalence fitted to Zimbabwe DHS prevalence estimates (red diamonds), with the time trend informed by the trends in prevalence in pregnant women tested in antenatal care (sentinel surveillance surveys, green symbols).


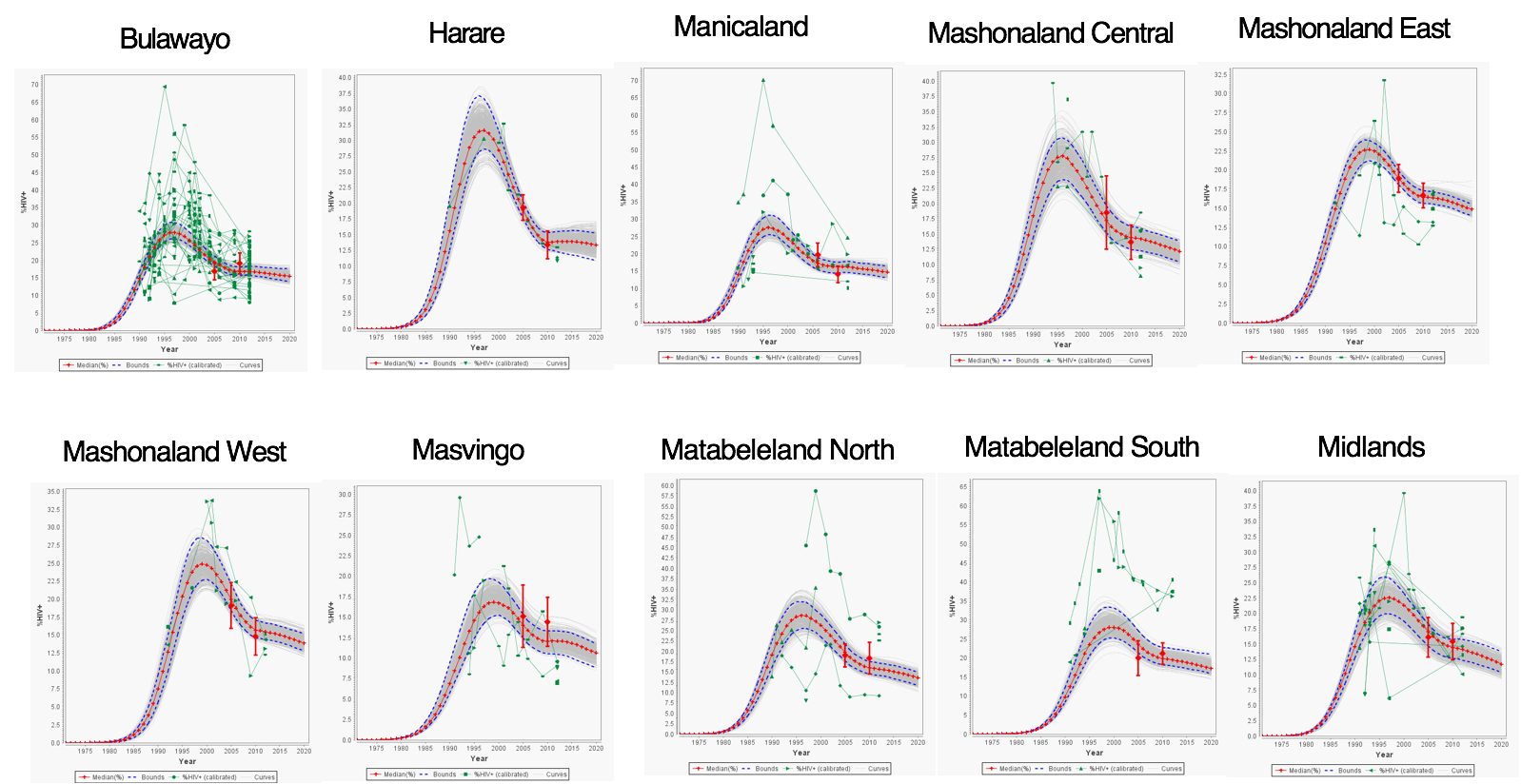


**Figure B. ICL model calibrations**: provincial-level HIV prevalence (among ages 15-49) produced by the calibrated model (black) in comparison with time trends from antenatal clinic surveillance (blue error bars), point estimates from the Zimbabwe 2015 DHS (blue squares), and the preliminary 2016 PHIA estimates (red diamonds).


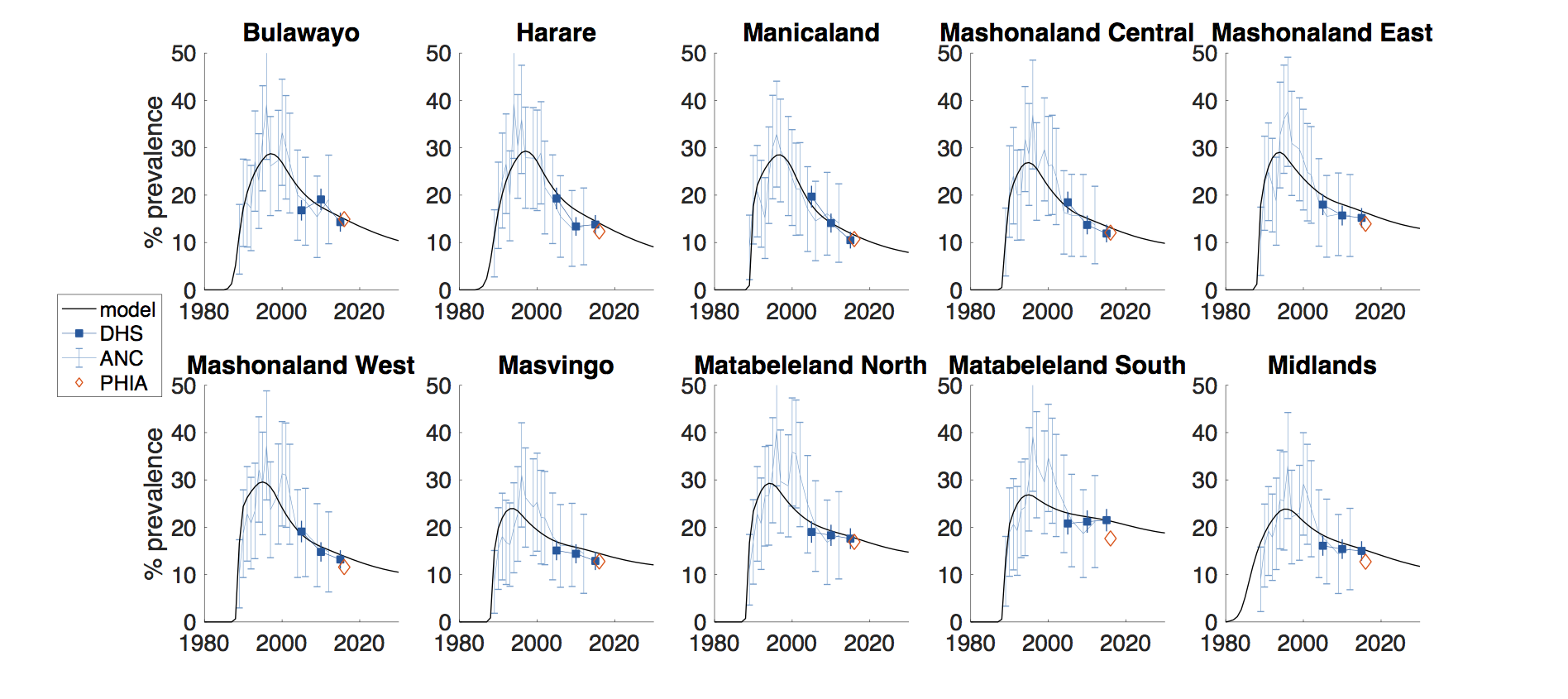


**Figure C. EMOD model calibrations**: model outputs (red) compared to data from DHS and ZIMPHA surveys (black). a) HIV prevalence by age, gender, and year. b) National prevalence over time for those aged 15-49, C) Percent of people living with HIV who have a suppressed viral load by age and gender from the 2015-2016 PHIA survey.

**
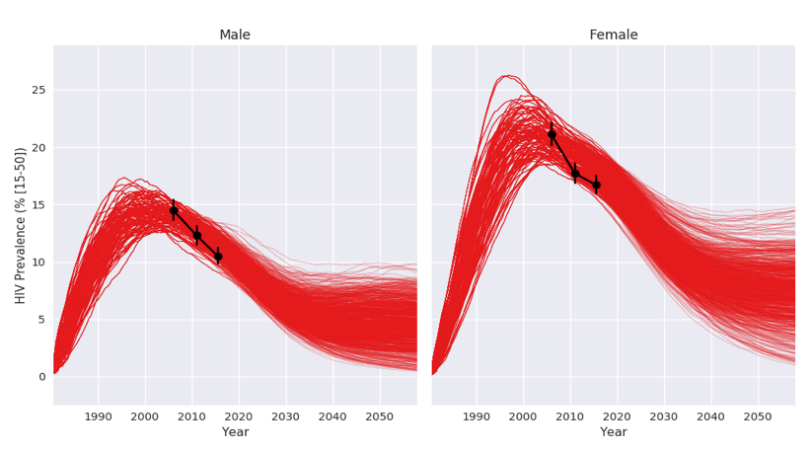
**a.


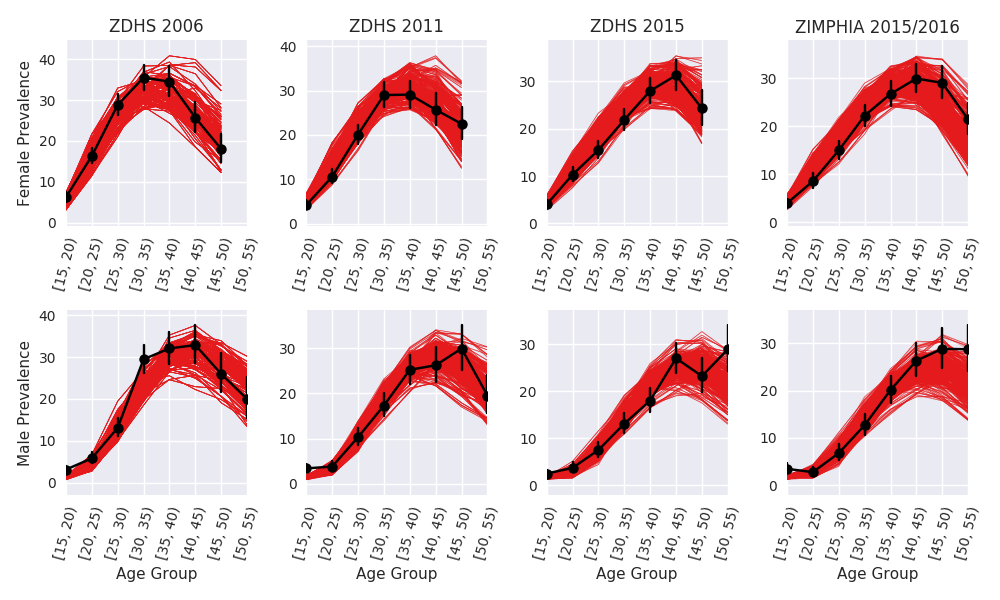
b.

c.

**
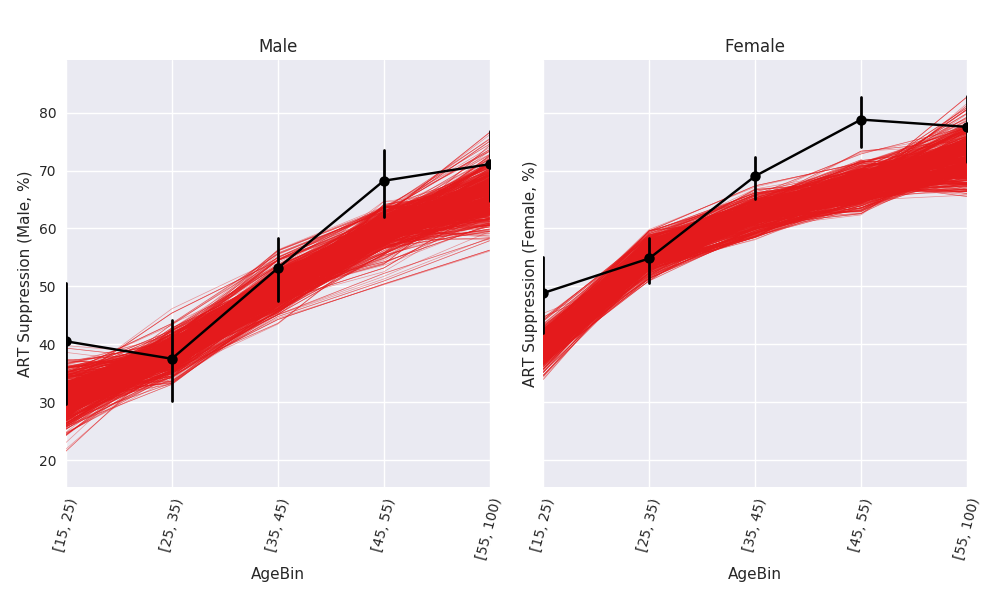
**

# C Assumptions for interventions in a 'Fast Track' modeling context

**Table A.** **Prevention and ART coverage targets** according to UNAIDS global Fast Track plan to end the HIV epidemic by 2030 [35]. We note that Fast Track additionally stipulates a 90% target for VMMC coverage among ages 10-29 for 14 priority countries in southern and eastern Africa, including Zimbabwe; however, for the Zimbabwe modeling presented here, we instead use the national (ASCOP) target of 80% among ages 15-29 and 30% among ages 10-14 across all scale-up scenarios.

| **Intervention** | **Target**  **2020** | **Target**  **2030** |
| --- | --- | --- |
| Prevention for key populations | 90% | 90% |
| Condom use with non-regular partners | 90% | 90% |
| PMTCT (Option B+) | 95% | 95% |
| Pre-exposure prophylaxis, for key (higher-risk) populations & sero-discordant couples | 10% | 30% |
| ART | 81% | 90% |

# D Key model differences

**Table B: Comparison of model structures** and estimation of impact

|  | **Goals** | **ICL** | **EMOD** | **Effect on modeled VMMC impact and cost-effectiveness** |
| --- | --- | --- | --- | --- |
| Age structure, within 15-49 years | No | No | Yes | Goals slightly overestimates impact and cost-effectiveness in the short term, and somewhat underestimates in the long term; ICL slightly underestimates in the short term. |
| Calculating infections (averted) among ages 0-15 and 50+ years | Yes | No | Yes | In Goals, in the scenario ‘VMMC program ends after 2016’, 92% and 91% of infections averted among all ages were in the 15-49 age group. ICL considers infections only among ages 15-49 and therefore may underestimate impact and cost-effectiveness by 8-9% relative to Goals. |
| Provincial or national-level epidemics | Provincial | Provincial | National | VMMC uptake has been (slightly) higher in provinces with lower HIV incidence, causing a slight overestimation in EMOD. |
| HIV status of men getting VMMC | Ignored | Only HIV negative men receive VMMC | Only HIV negative men receive VMMC | Goals slightly underestimates impact, because it attributes 11% of VMMCs to HIV-infected men, whereas in reality 99% of VMMCs went to HIV-uninfected men. |
| Population and transmission structure | Groups / compartments | Groups / compartments | Individuals / network | Could go in either direction; EMOD is more valid if well-calibrated on valid data. |
| MSM in the HIV epidemic | Ignored | Across provinces, 3.2% of men 15-49 years are MSM; in 2016, 6.5% of new infections among men 15-49 years were in MSM; 0% of VMMC uptake is among MSM (and VMMC does not reduce susceptibility to new infection among MSM) | Ignored | Goals and EMOD slightly over-estimate impact, since VMMC effectiveness is less for MSM than for heterosexual contacts. ICL slightly under-estimates impact by assuming 0 VMMC effectiveness and uptake in MSM. |

# E Alternative estimate for baseline MC coverage in 2016

Our best models and estimates had assumed that MC coverage, as a result of traditional MCs at a fixed level as at 2008 pre-program (Figure D, lightest-green bars), plus from VMMCs added through the VMMC program (S2 table), reached 28% nation-wide by 2016 (Figure D, red bars). In contrast, self-reports by men 15-49 years in the 2015 DHS and PHIA 2016 suggested a considerably lower coverage of only 14.3% (Figure D, green and blue bars).

**Figure D. Alternative circumcision coverages** among men ages 15-49 years, as measured in four household surveys and compared with our estimates based on program VMMC delivery statistics. "Mash" = Mashonaland, "Mat" = Matabeleland. The survey data are sourced from the DHS in 2005/6 [29], 2010/11 [30], 2015 [51], a 2014 multiple indicator cluster survey [55], and the 2016 Zimbabwe Population-based HIV impact assessment (PHIA) [7].


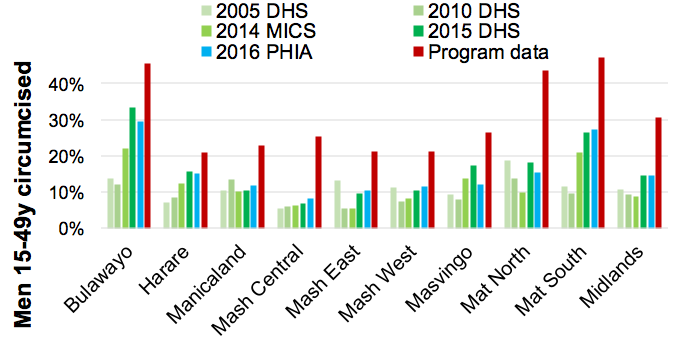


Many factors could be contributing to this difference. Respondents in the 2015 DHS and 2016 PHIA may have underreported MCs, particularly traditional circumcisions, or mis-reported traditional MCs as VMMCs. Replacement of traditional MCs by VMMCs may, in theory, be a contributor to the difference; however, this is unlikely to be the case in Zimbabwe, since traditional MC rates were low and the program service delivery statistics suggest MC rates much higher than the surveys among older-aged-men, while few traditional circumcisions take place among men over 25 years. Both the 2015 DHS and 2016 PHIA asked respondents if they received a medical or non-medical circumcision, and reported rates of medical circumcisions are high: in the PHIA, 11.8% and 2.3% of men overall reported medical and non-medical circumcision, respectively, and the corresponding rates among men 45-49 years were 5.8% and 5.1%. Since these older men were above age 30 before the VMMC program began, they must have been reporting non-VMMC circumcision as medical.

The 2015 DHS and 2016 PHIA may also have under-sampled VMMC target groups, such as military conscripts, although this would not explain the mismatch for older-aged men. For the 2015 DHS specifically, a small part of the discrepancy may be explained by the fact that the DHS was conducted at the end of 2015 and we have used programmatic results up to the end of 2016; however, this does not explain the mismatch between the program data and the 2016 PHIA. Migration, particularly to and from South Africa, may have affected both survey participation and the size of the target population for the VMMC program. Lastly, there may be over-reporting or double-counting errors in the program data, although Zimbabwe has conducted regular data quality audits and on-site data verifications since 2015, which have generally confirmed the accuracy of VMMC service delivery statistics among men who were traced for follow-up.

In summary, we do not quite understand the reasons for the discrepancy, and therefore here present the key modeling results in an alternative calibration, running all three models with the 'baseline' 2016 circumcision coverage based on the 2016 MC coverage according to the survey reports rather than the program data. The Results section of the main text (sensitivity analysis sub-section, including Table 4) presents key impact and cost-effectiveness results from this sensitivity analysis. Below are additional detailed and intermediate results from these model variants.

**Table C. Impact over 2009-2016** of the VMMC program with 2016 MC coverage calibrated to the 2015 DHS and 2016 PHIA data, rather than VMMC program statistics used in the best/default scenario. The percentages in parentheses represent the percentage of new HIV infections averted, relative to the total number of new infections in the counterfactual (no VMMC ever) scenario. As with the tables presented in the main text, outcomes are among all ages in Goals and EMOD and among ages 15-49 only in ICL.

|  | **Goals**^a^ | **ICL**^b^ | **EMOD**^a,c^ |
| --- | --- | --- | --- |
| Number of VMMCs over 2009–2016 | 423,000 | 450,000 | 223,000 |
| Number of HIV infections averted | 3,100 (0.6%) | 3,500 (0.8%) | 250 (0.05%) |
| Number of VMMCs per infection averted | 136 | 126 | 920 |
| Cost per infection averted | $14,800 | $13,725 | $100,000 |

^a^ Health and cost-effectiveness outcomes are among all ages

^b^ Health and cost-effectiveness outcomes are among ages 15-49 years only

^c^ Over this short time period, EMOD outcomes are influenced by noise from stochastic variation

**Table D. Impact over 2009-2030** of the VMMC program (with and without continuation after 2016) with 2016 MC coverage calibrated to the 2015 DHS data, rather than the VMMC program statistics used in the best/default scenario. The percentages in parentheses represent the percentage of new HIV infections averted, relative to the total number of new infections in the counterfactual (no VMMC ever) scenario.

| **Scenario** | **Outcome** | **Goals**^a^ | **ICL**^b^ | **EMOD**^a^ |
| --- | --- | --- | --- | --- |
| Program ends after 2016 | Number of VMMCs | 423,000 | 450,000 | 223,000 |
|  | Number of HIV infections averted | 22,000 (1.7%) | 12,000 (1.1%) | 11,500 (1%) |
|  | Number of VMMCs per infection averted | 19 | 37 | 19 |
|  | Cost per infection averted | $2,100 | $4,000 | $2,100 |
| Program targets through 2021 met & maintained | Number of VMMCs | 3,197,000 | 2,302,000 | 3,165,000 |
|  | Number of HIV infections averted | 156,000 (12%) | 120,000 (11%) | 104,500 (8.5%) |
|  | Number of VMMCs per infection averted | 20 | 19 | 30 |
|  | Cost per infection averted | $2,200 | $2,100 | $3,300 |

^a^ Health and cost-effectiveness outcomes are among all ages

^b^ Health and cost-effectiveness outcomes are among ages 15-49 years only.

References (continued from main text)

1. Lodi S, Phillips A, Touloumi G, Geskus R, Meyer L, Thiébaut R, Pantazis N, Amo JD, Johnson AM, Babiker A, Porter K. Time from human immunodeficiency virus seroconversion to reaching CD4+ cell count thresholds <200, <350, and <500 cells/mm3: assessment of need following changes in treatment guidelines. *Clin Infect Dis* 2011; 53(8):817-25.
2. Bellan SE, Dushoff J, Galvani AP, Meyers LA. Reassessment of HIV-1 acute phase infectivity: accounting for heterogeneity and study design with simulated cohorts. *PLOS Med* 2015; 12(3): e1001801.
3. Andrianakis I, Vernon IR, McCreesh N, McKinley TJ, Oakley JE, Nsubuga RN, Goldstein M, White RG. Bayesian history matching of complex infectious disease models using emulation: a tutorial and case study on HIV in Uganda. *PLOS Comput Biol* 2015; 11(1):e1003968.
4. United Nations Department of Economic and Social Affairs. The world population prospects: 2015 revision. New York, USA, 2015. URL: http://www.un.org/en/development/desa/publications/world-population-prospects-2015-revision.html [Accessed 18 Dec 2017].
5. Zimbabwe National Statistics Agency (ZIMSTAT). Zimbabwe Demographic and Health Survey 2015. Harare: Government of Zimbabwe (GovZWE), United States Agency for International Development (USAID), United Nations Population Fund (UNFPA), United Nations Development Programme (UNDP), 2015.
6. Schur N, Mylne A, Mushati P, Takaruza A, Ward H, Nyamukapa C, Gregson S. The effects of household wealth on HIV prevalence in Manicaland, Zimbabwe—a prospective household census and population-based open cohort study. *J Int AIDS Soc* 2015; 18: 20063.
7. Lopman B, Nyamukapa C, Mushati P, Mupambireyi Z, Mason P, Garnett GP, et al. HIV incidence in 3 years of follow-up of a Zimbabwe cohort—1998-2000 to 2001-2003: contributions of proximate and underlying determinants to transmission. *Int J Epidemiol* 2008; 37(1): 88-105.

1. Chingandu L. Multiple concurrent partnerships: the story of Zimbabwe—are small houses a key driver? SAfAIDS.
2. UNICEF, Zimbabwe National Statistics Agency. Zimbabwe—Multiple Indicator Cluster Survey 2014. Harare, Zimbabwe, 2014. URL: http://microdata.worldbank.org/index.php/catalog/2527 [Accessed 19 January 2018].
